# Marginal and Internal Fit of CAD/CAM and Slip-Cast Made Zirconia Copings

**DOI:** 10.5681/joddd.2012.010

**Published:** 2012-06-06

**Authors:** Kianoosh Torabi Ardekani, Ahmad Hassan Ahangari, Leila Farahi

**Affiliations:** ^1^Associate Professor, Department of Prosthodontics, School of Dentistry, Shiraz University of Medical Sciences, Shiraz, Iran; ^2^Assistant Professor, Department of Prosthodontics, School of Dentistry, Shiraz University of Medical Sciences, Shiraz, Iran; ^3^Resident, Department of Prosthodontics, School of Dentistry, Shiraz University of Medical Sciences, Shiraz, Iran

**Keywords:** CAD/CAM, internal fit, marginal fit, slip-cast, zirconia

## Abstract

**Background and aims:**

CAD/CAM systems facilitate the use of zirconia substructure materials for all-ceramic copings. This in vitro study investigated the marginal and internal fit of zirconia copings made with CAD/CAM system and slip-casting method.

**Materials and methods:**

Sixteen CAD/CAM made zirconia copings and 16 slip-cast made zirconia copings were fabri-cated and cemented with glass-ionomer cement to their respective master abutment models, and thickness of the cement layer was measured at specific measuring points with stereomicroscope.

**Results:**

In the left wall, the mean axial internal gap was greater in group one than group two (62.49 vs. 48.14) (P =0.007), in the right wall the mean axial internal gap was greater in group one than group two (44.87 vs. 40.91) (P = 0.465). The oc-clusal internal gap was greater in group one than group two (118.81 vs. 102.11) (P =0.423). The mean marginal gap also was greater in group one than group two (46.67 vs. 44.29) (P = 0.863). The differences in marginal fit between these two methods were not statistically significant, except for left axial internal gap that was significantly greater in the CAD/CAM system than conventional slip-cast technique (P =0.007).

**Conclusion:**

It was concluded that this CAD/CAM system can compete well with conventional systems for clinical fit, and can achieve good in vitro marginal fit.

## Introduction

In order to meet patients' expectations to achieve good esthetic results, and biocompatibility, and also their concerns about allergy due to contacts with metallic frameworks,^1^ all-ceramic restorations have become both a necessary alternative and a preferred choice.

All-ceramic restorations have been successfully used for restoring anterior as well as posterior teeth.^2^ In the construction of all-ceramic crowns, similar to metal ceramic crowns, a high strength ceramic coping is used in order to resist against loading. In addition to resistance to fracture and esthetics, one of the most important criteria for the clinical quality and success of all-ceramic crowns is the marginal and internal accuracy of fit.^3^

When the marginal discrepancies are great, the cement material is exposed to the oral environment, and this leads to a higher rate of cement dissolution, which is caused by oral fluids and chemo-mechanical forces. When the cement seal is weakened, the percolation of bacteria is the result.^4^ Consequently, the longevity of the teeth could be placed in danger by caries and periodontitis. Misfit in the axial wall area and occlusal plateau can also reduce the resistance to fracture of all-ceramic restorations.^5^

Different high-strength ceramic materialsand various constructing methods may be used to make ceramic crown-copings.^6^

In-Ceram Zirconia is a glass-infiltrated zirconia-toughened alumina (ZTA), in which, for the first time, zirconium oxide was used as in a dental ceramic.^7^ Its flexural strength is 400–800 MPa. Yttrium partially stabilized tetragonal zirconia polycrystal (3Y-TZP), is the strongest and most commonly used zirconia-based ceramic.^8 - 11^

3Y-TZP, which is characterized by a flexural strength of approximately 900 Mpa and a fracture toughness of 9 Mpa/m ^2 ^, is more suitable than all other presently available all-ceramic framework materials.^12^

One method for fabricating zirconia copings and frameworks is computer-aided design/ computer aided manufacturing (CAD/CAM). CAD/CAM fabrication of zirconia copings involves the manipulation of a 3D design on the computer screen, followed by the automated production by a computer-controlled milling machine.^13^

CAD/CAM zirconia dental frameworks can be produced according to two different techniques: “soft machining” of presintered blanks or “hard machining” of fully sintered blanks. ^8, 14, 15^

Using CAD/CAM technology has significant advantages because of the fact that room temperature milling of ceramic materials which have gone through high-quality processes will yield homogenous materials structures, where voids, flaws, and cracks are reduced to a minimum.^16^

Therefore, CAD/CAM manufacturing of all- ceramic restorations from an industrially prepared ceramic block can be seen as an alternative technique for fabrication of dental restorations.^17^

For practical use, the obvious efficiency of the CAD/CAM method had to be evaluated against potential inaccuracies due to the scanning process, software design, milling, and shrinkage effects. These inaccuracies could lead to poor restoration fit.^18^

Based on the above mentioned information we arrived at the hypothesis that the accuracy of fit of copings produced with the CAD/CAM system used in this study, Tizian CAD/CAM system (Schutz Dental GmbH, Rosbach, Germany) is similar to that produced using conventional slip-casting technique.

The purpose of this in vitro study was to investigate the marginal and internal fit of conventionally and CAD/CAM manufactured all-ceramic zirconia copings.

## Materials and Methods

Thirty two master abutments (model) were designed and prepared by CNC milling machine (CNC350, Arix Co. Tainan Hesin, Taiwan). These models were made from brass alloy blocks. Figure 1 (c & f) illustrates the shape and dimension of the master abutment.

The amount of reduction of each model was measured with a digital caliper (Mitutoyo Corp, Kawasaki, Japan). The metal dies were polished and finished with a CNC milling machine (T3; Degussa AG, Hanau, Germany). The prepared models were seen under a light microscope (Leica DM3000, Leica Microsystem Corp, Wetzlar, Germany) with a magnification of ×12.5 for final observation before coping construction.

For taking impressions, each master abutment was fixed on a plastic plate with cianoacrylate and placed in dental stone type III (DS1, Guangzhou Gypsum Products Comp, China) which half of the base of each model was embedded in stone for better stability. Impressions of each model were taken using additional silicone impression material (Genie, Sultan Chemist Inc, Englewood, England). An American dental Association (ADA) type IV die stone (Fujirock EP, GC Corp, Tokyo, Japan) was then cast in the impressions to produce stone models.

A dental CAD/CAM system, Tizian CAD/CAM system (Schutz Dental GmbH, Rosbach, Germany) was used to fabricate the 16 zirconia copings used in this study. Stone models of the abutments were scanned using Tizian CAD/CAM Opt Scanner. Scanned data were then converted into CAD data. Copings for all-ceramic crowns were designed using the CAD/CAM Creativ RT software. For the CAD/CAM system used in this study, the amount of cement space at the margin, axial and occlusal surface could be determined independently when designing the copings. No cement space was included for the margin, and 45 µm was used for the axial and occlusal surfaces of the abutment. Thickness of the copings were designed to be 0.7 mm. Design data were converted into processing data and sent to the processing machine.

Designed copings were milled with burs of 1 and 3 mm diameter from raw stage zirconia blanks of partially stabilized zircon powder (zirconium dioxide < 96%, yttrium oxide > 4%, hafnium oxide > 1%, aluminum oxide < 1%, silicone oxide < 0.02%) (% weight) mixed with a binder.


Figure 1. (a) Marginal gap measurement: example of a microscope picture (magnification ×40) for measuring marginal and internal gap widths. (b) measurement locations for marginal and internal gap (magnification ×7); (c) master abutment with its antirotational part; (d) cross-section of master abutment and its coping embedded in aluminum block; (e) Coping immediately after cementation under 50 N in universal testing machine; (f) shape and dimension of different parts of the coping.

**a:**
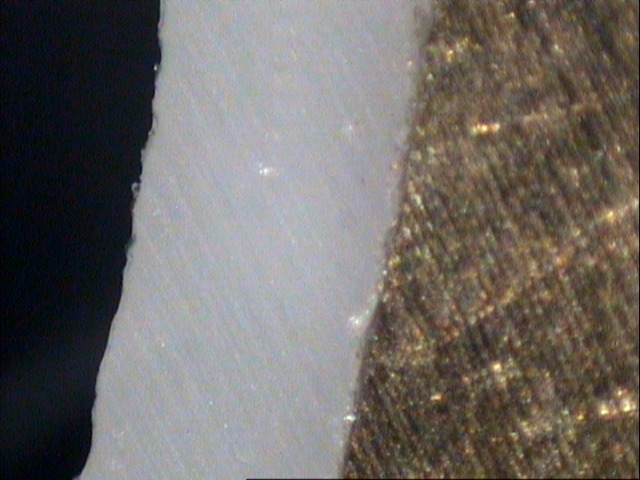

**b:**
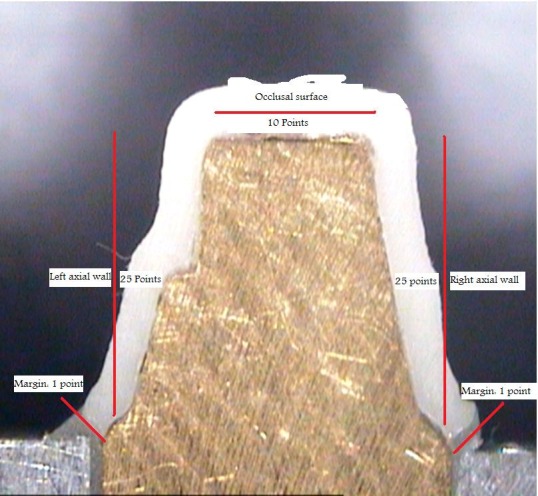

**c:**
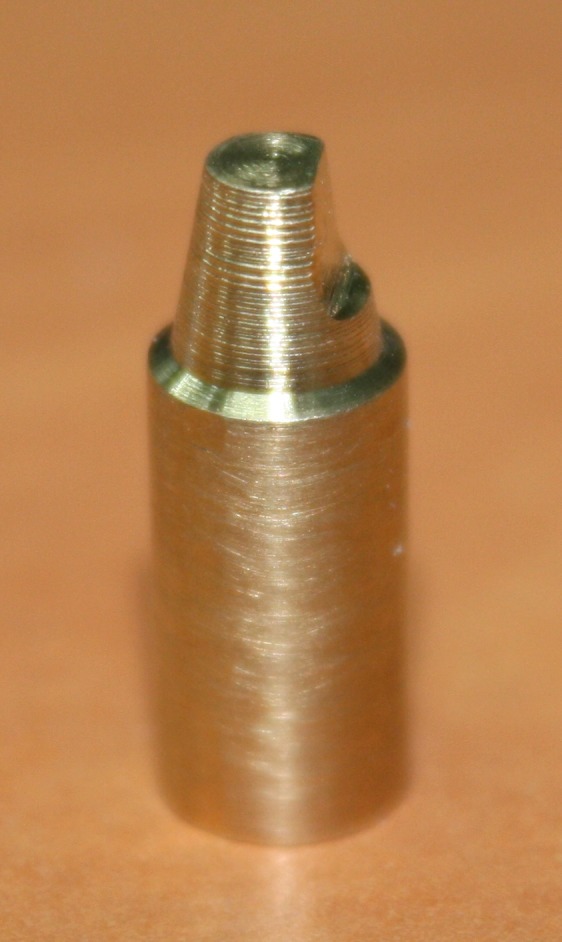

**d:**
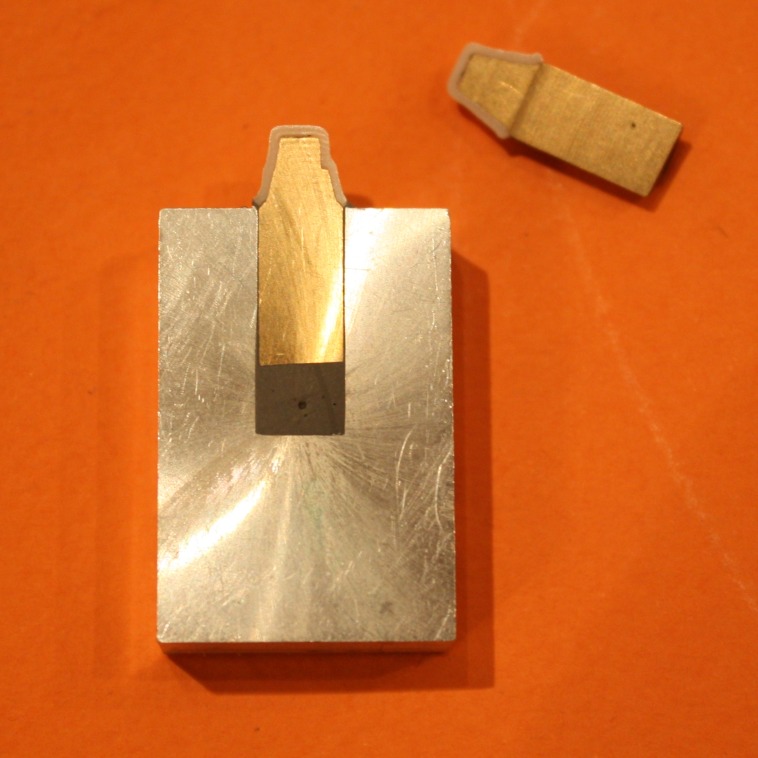

**e:**
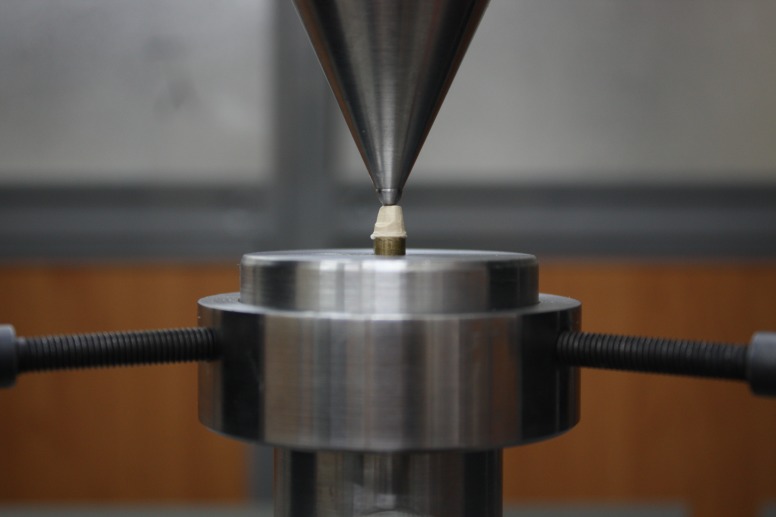

**f:**
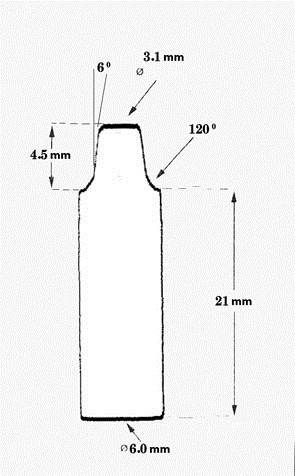

Prior to dense sintering, the copings were freed from dust caused by grinding. The copings milled approximately 25% greater in size, were shrunk to their original size by dense sintering in Tizian Furnace S (Start point: room temperature, heating rate: 8°C/min, final temperature: 1450°C). The copings obtained the desired physical properties through sinter firing.

Another 16 zirconia copings used in this study were made using conventional slip-casting technique.

Copings that could be rotated on the master abutments were rejected and new copings were fabricated on the same models. By using silicone pressure indicator paste (fit checker, GC Corp, Coltene, Switzerland) pressure spots were removed from the internal surface of the copings. Then, complete zirconia copings were cemented to each master abutment model using Glass Ionomer cement (KetacCem Easymix; 3MESPE, US) according to manufacturer’s specifications and the excess cement was removed by a disposable brush.

Cemented pieces were immediately subjected to a fixed load of 50 N^19^for 10 minutes with universal testing machine (Zwick-Roell Z020, Zwick GmbH & Co. KG, Ulm, Germany) (Figure 1e). Before embedding cemented copings with their respective master abutment models in Aluminum blocks (Figure 1d), they were assessed with a light microscope (Leica DM3000, Wetzlar, Germany) and the largest gaps at the margin (or in fact the thickest cement material) were marked at 2 or 3 points in order to use these points as a reference where the sectioning line will pass from at least one of these points.

The sectioning machine was a low speed diamond cutting machine (Isomet # 4000-5000, Buehler Ltd., Lake Bluff, IL, USA). Thickness of the cement layer was measured using a stereomicroscope (Leica M80, Heerbrugg, Switzerland) at ×40 magnification (Figure 1a).

The cross-sections were adjusted horizontally on modeling clay in order to obtain a parallel orientation to the microscope’s plate and to achieve a vertical observation angle.

The measurements were done via a personal computer, attached to a stereomicroscope (Leica M80, LeicaSolms microsystem, Heerbrugg, Switzerland) by using Intervideo Software and a camera (Leica IC80, LeicaSolms microsystem, Heerbrugg, Switzerland). The camera produced an image of the cross-section on computer monitor. The discrepancies were measured by moving a cross from one end to the other end of a measuring distance. The distance was counted in microns on a screen with the size of 640×480 pixels.

The procedure was carried out by one trained investigator, who was not involved in this study. At each cross-section 62 points were measured:

Two points for marginal area, 50 points for axial surfaces, (each of them 25 points), 10 points for occlusal surface (Figure 1b).

It should be mentioned that all of the sections were passed through antirotational part of the master abutments because at least one point of greatest marginal gap that was marked under light microscope was at the same plane with this part. So, the axial wall that was consisted of the antirotational part of the master abutment was called left axial wall and the other one, right axial wall.

The statistical analysis was carried out using SPSS software. Descriptive statistics included the calculation of the mean, standard deviation (SD), median, minimum, maximum and range of all available measurements for each surface. The independent t-test was used to evaluate the differences between internal and marginal gaps of the coping made with these two different methods.

## Results

In the copings made with CAD/CAM system (group one), the mean axial internal gap was 62.49 for left wall 44.87 for right wall and 53.74 for mean of both of axial walls.

**Figure 2:**
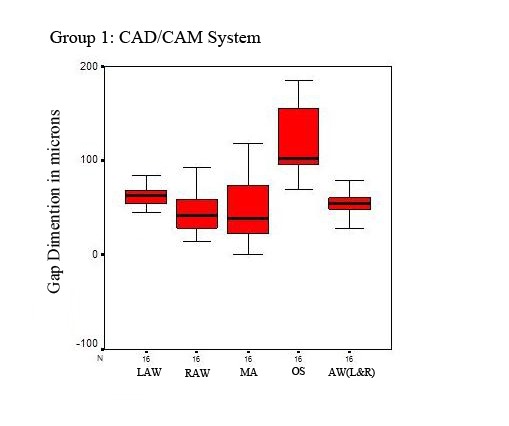
Box plot of the marginal and internal gap measurements in group 1 (CAD/CAM system)

The mean occlusal internal gap was 118.81 and the mean marginal gap was 46.67 (Table 1, and Figure 2).

**Table 1:** Mean, median, minimum, maximum, and standard deviation of gap widths at four measuring areas

Group		MG	RAW	LAW	OS	AW (L & R)
1	Mean Number Std. Deviation Median Minimum Maximum Range Variance	62.4858 16 13.83291 62.8080 33.83 83.89 50.06 191.349	44.8664 16 19.40660 41.8180 14.14 92.11 77.97 376.616	46.6658 16 35.18470 38.8520 0.00 118.29 118.29 1237.963	118.8147 16 35.90704 101.4500 69.27 184.27 115.00 1289.315	53.7441 16 12.32292 54.3400 28.94 78.28 49.34 151.854
						
2	Mean Number Std. Deviation Median Minimum Maximum Range Variance	48.1409 16 13.07986 46.5355 22.64 71.40 48.76 171.083	40.9097 16 17.22067 39.7865 21.77 81.85 60.09 296.551	44.2859 16 33.35940 29.7920 0.00 123.65 123.65 1112.850	102.1131 16 31.61686 106.3550 24.12 152.58 128.46 999.626	45.2787 16 13.47152 44.5150 22.20 76.62 54.42 181.482

LAW: Left axial wall; RAW: right axial wall; MA: marginal area; OS: occlusal surface; AW (L & R): axial wall left and right.

In the coping made with conventional slip-cast technique (group two), the mean axial internal gap was 48.14 for left wall, 40.91 for right wall, and 45.28 for mean of both of axial walls.

**Figure 3:**
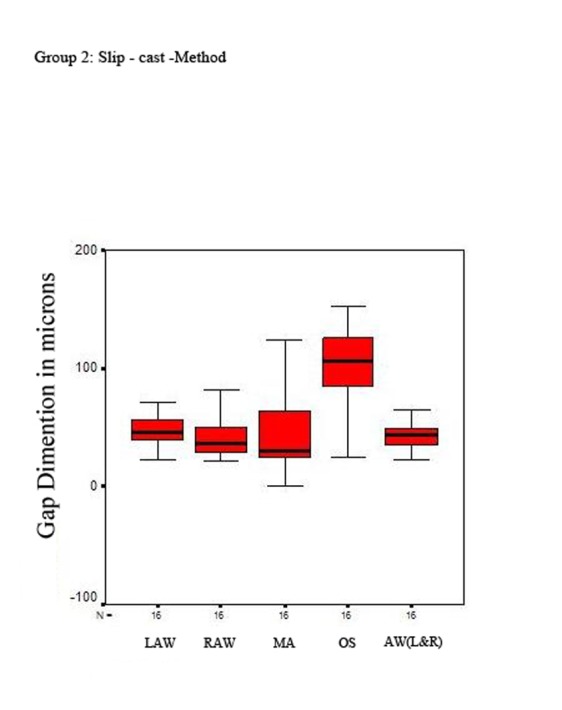
Box plot of the marginal and internal gap measurements in group 2 (slip-cast method).

The mean occlusal internal gap was 102.11 and the mean marginal gap was 44.29 (Table 1, and Figure 3).

In the left wall, the mean axial internal gap was greater in group one than group two (62.49 vs. 48.14) (p = 0.007); in the right wall, the mean axial internal gap was greater in group one than group two (44.87 vs. 40.91) (p = 0.465). The occlusal internal gap was greater in group one than group two (118.81 vs. 102.11) (p = 0.423). The mean marginal gap was also greater in group one than group two (46.67 vs. 44.29) (p = 0.863).

## Discussion

It was found that the marginal gap of copings were almost the same as those of the designed cement space but the thickness of the cement layer on the axial surface of copings were greater than the value set by the software (45 µm). Compared with values reported previously using other CAD/CAM systems, these values were excellent.^20 , 21^

On the other hand, the mean value of the thickness of cement layer at the occlusal surface was larger than the value set by the software. It is assumed that this large discrepancy could be attributed to the anisotropic shrinkage of zirconia blanks subjected to post-machining sintering.

The differences in marginal fit between these two methods were statistically not significant except for left axial internal gap that was greater in the CAD/CAM system than conventional slip-cast technique (p = 0.007). This gap may be attributed to inaccuracies created during scanning process on “not at the same plane surfaces” (left axial wall) compared with surfaces that are at the same plane (right axial wall). The relevance of these differences in clinical setting is in doubt, because the mean marginal gap values and even the maximum value of these two methods were below the recommended clinical limit of 120 µm.^4 , 22^

Thin cement layers (80 µm) at occlusal surface have been reported to be more favorable for the mechanical stability of zirconia based restorations.^23^ One in-vitro study showed that a lack of precision in internal fit may promote higher risks for veneering fracture.^24^ The observation that the fit of CAD/CAM generated crowns is less accurate in the internal regions than that of the margin area, confirms the findings of earlier studies.,^25^

Using the software, the point clouds obtained in scanning area were transformed into a smooth, continuous surface, and this can lead to internal inaccuracies.^26^ The grinding process and the preparation design may also affect the internal adaptation. The narrowest possible diameter of the preparation is determined by the smallest diameter of the bur used for machining the internal surface. Thus, in structures smaller than the narrowest bur diameter, more internal substance may be removed than necessary. This may also result in larger than mandatory internal gaps for a good fit. ^25, 27^

A source of error is the wear of milling instruments during milling. During the milling procedure diamond grains area plucked out, changing the radius of the instruments and reducing milling precision. A change of the milling instruments at regular intervals is highly recommended to control this factor.^3^

Apart from the mechanical properties of the material used, the internal fit also has a practical aspect. If too much space is lost as a result of large interocclusal discrepancies, the interocclusal clearance available for veneering is reduced.^18^

Steel dies or resin dies have been employed by several authors for measurements of the marginal accuracy. The advantage of this method is the possibility of a standardized preparation for all abutments. However, abutments made of steel or resin, give neither real information about the microstructure of the hard tissue of the teeth after preparation nor about the micro- and chemo- mechanical adaptation of the luting material to the dentin.^6^

Since the coping mainly determines the overall fit of a veneered crown, the fit of the copings was measured without veneering in this study.^4 , 6^ This study therefore was focused on the differences between the coping fabrication processes.

It has to be considered that this study used the cross- sectional technique to obtain the data. This technique might lead to a lack of information concerning the precision of fit. It might be questioned if the measured areas represented the precision of fit of the whole specimen. However, several studies used the cross-section technique to evaluate the precision of fit.^4 , 18 , 20 , 28 , 29^

The results show that the Tizian CAD/CAM system is able to calculate the sintering shrinkage of the zirconia green blanks successfully within the indications mentioned in this study. The use of a green blank offers the advantages of an easy machining process. The block can be machined with a hard metal bur without water cooling or lubrication.^18^

The copings were fabricated under optimal laboratory conditions. Clinically, the fit of all-ceramic restorations is influenced by other factors such as tooth preparation, impression technique and cementation methodology, which have not been evaluated in the present study. Another limitation of this study is that the copings were not subjected to an artificial aging process.

The precision of the zirconia-based restorations is dependent on various factors, like differences in manufacturing systems, individual characteristics of the prosthesis (e.g. span length, framework configuration), effect of veneering and influence of aging. As to soft-machined 3Y-TZP restorations, the precise numerical compensation required by such a system for the enlargement ratio of the model is an important factor, also dependent on the composition and homogeneity of pre-sintered zirconia blanks that should be consistent and precise.

## Conclusion

We conclude that within the limitations of this study, this CAD/CAM system can compete well with conventional systems for clinical fit, and can achieve good in-vitro marginal and internal fit.
